# Experimental and simulation data for point-by-point wire arc additively manufactured carbon steel bars loaded in uniaxial tension

**DOI:** 10.1016/j.dib.2024.110093

**Published:** 2024-01-24

**Authors:** Vlad-Alexandru Silvestru

**Affiliations:** Institute of Structural Engineering, ETH Zurich, Stefano-Franscini-Platz 5, 8093 Zurich, Switzerland

**Keywords:** Wire arc additive manufacturing, Uniaxial tensile test, Imperfect geometry, Elastic-plastic material properties, Finite element simulation

## Abstract

Wire arc additive manufacturing is considered to allow a reduced material consumption for structural steel components by efficiently distributing the material only where necessary. Parts produced with this technology exhibit an irregular, imperfect geometry, which influences their structural behaviour. This paper describes a dataset, which includes geometry information for point-by-point wire arc additively manufactured steel bars, force and displacement measurements from performed uniaxial tensile tests on such bars, and force and displacement values from geometrically and materially non-linear simulations of the bars with imperfect geometry. The geometry data was obtained by 3D scanning the steel bars. Moreover, a script is provided that allows processing the scanned geometry data such that it can be used to generate suitable finite element meshes for geometrically and materially non-linear analyses. The force and displacement data from the uniaxial tensile tests were collected through measurements with a load cell for the force and with the help of digital image correlation measurements for the displacements. The non-linear simulations of the experiments were conducted with the computer aided engineering software Abaqus on processed approximations of the irregular scanned geometry. The described dataset can be used for better understanding the influence of the irregular geometry on the structural behaviour of wire arc additively manufactured parts. Moreover, researchers can apply the data to validate finite element simulation models and approaches for predicting the structural behaviour of different wire arc additively manufactured parts.

Specifications Table

**Table:** 

Subject	Civil and Structural Engineering
Specific subject area	Structural Behaviour of Wire Arc Additively Manufactured Steel Components in laboratory tests and finite element simulations
Data format	Raw, Analysed
Type of data	1) Geometry of the wire arc additively manufactured test specimens (mesh in STL file format, points in CSV file format); 2) Table with force and displacement values from the experiments (CSV file format); 3) Grasshopper script for generating the simplified specimen geometry for the finite element simulations (GH file format); 4) Simplified geometry of the test specimens for finite element simulations (mesh in STL file format, points in CSV file format, closed surface in STP file format); 5) Input files for the finite element simulations (text files in INP file format) 6) Table with force and displacement values from the finite element simulations (CSV file format).
Data collection	The geometry of the wire arc additively manufactured test specimens was obtained by 3D scanning with a GOM ATOS Core instrument. The obtained irregular meshes were processed with a Grasshopper script to generate simplified closed surfaces that can be used for finite element simulations. The wire arc additively manufactured specimens were tested in uniaxial tension on a Zwick universal testing machine under displacement control. The displacements were obtained from digital image correlation measurements. The finite element simulations were performed geometrically and materially nonlinear with the computer aided engineering software Abaqus 2021.
Data source location	Institution: ETH Zurich, Institute of Structural Engineering City: Zürich 8093 Country: Switzerland
Data accessibility	Repository name: Dataset for point-by-point wire arc additively manufactured carbon steel bars loaded in uniaxial tension - experiments and simulations Data identification number: https://doi.org/10.3929/ethz-b-000639004 Direct URL to data: https://www.research-collection.ethz.ch/handle/20.500.11850/639004
Related research article	V.-A. Silvestru, I. Ariza, J. Vienne, L. Michel, A.M. Aguilar Sanchez, U. Angst, R. Rust, F. Gramazio, M. Kohler, A. Taras, 2021. Performance under tensile loading of point-by-point wire and arc additively manufactured steel bars for structural components, Mater. Des. 205, 109740. https://doi.org/10.1016/j.matdes.2021.109740.

## 1 Value of the Data

• These data are useful in understanding how the irregular and imperfect scanned geometry of test specimens in general, and wire arc additively manufactured test specimens in particular, can be considered in finite element simulations.
• The data is useful in validating finite element simulation models and approaches for predicting the structural behaviour of wire arc additively manufactured parts.
• Researchers in the field of wire arc additive manufacturing in general, and especially those dealing with wire arc additive manufacturing in structural engineering may benefit from this dataset for their own research related to the influence of the imperfect irregular geometry of wire arc additively manufactured parts on their structural behaviour.
• The geometry data can be analysed with different methods to evaluate geometry-related parameters of wire arc additively manufactured specimens.
• Researchers can use the experimental and simulation data to validate their finite element models for predicting the structural behaviour of wire arc additively manufactured parts.

## 2 Background

Wire arc additively manufactured parts exhibit irregular surface geometries. For characterising such surfaces, generally 3D scanning is used to obtain a point cloud or a mesh that can be further processed and analysed by various mesh-processing software. The meshes obtained from the 3D scanning are highly irregular and not suitable for generating finite element meshes for simulations. A more regular mesh needs to be generated to approximate the 3D scanned one. These mesh-processing steps were performed in the related research article for wire arc additively manufactured steel bars with a script in the Rhino3D/Grasshopper environment. After the related research article was published, the corresponding author received several inquiries on how the geometry for finite element simulations of wire arc additively manufactured parts can be generated from the 3D scanned mesh. This interest in the methodology used to process the 3D scanned mesh for use in finite element simulations represented the original motivation for compiling the dataset described in this data article.

## 3 Data Description

**Fig. 1:**
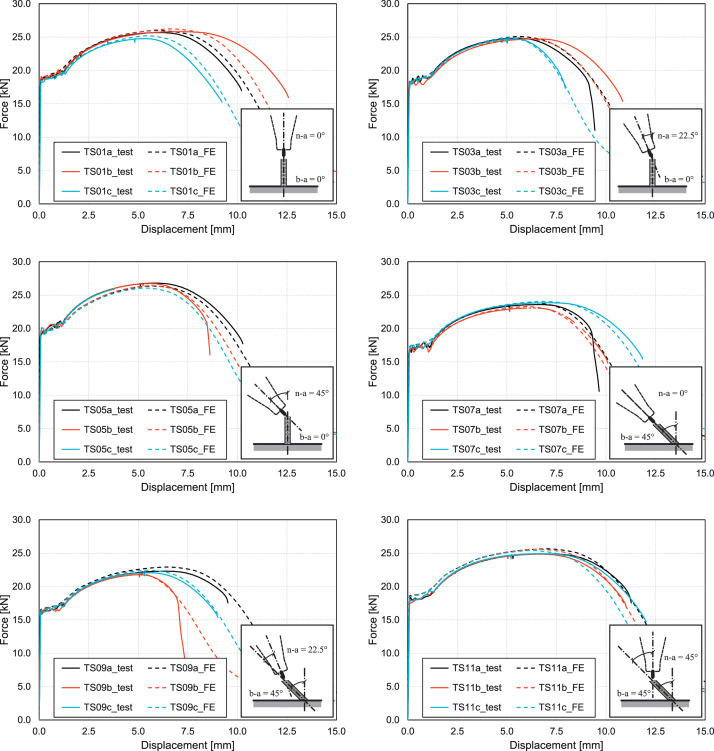
Force versus displacement data from the uniaxial tensile tests and the corresponding finite element simulations displayed as curves for the different test series listed in Table 1.

The data presented in this article includes geometry information for point-by-point wire arc additively manufactured steel bars, force and displacement measurements from performed uniaxial tensile tests on such bars, and force and displacement values from geometrically and materially non-linear simulations of the bars with imperfect geometry. Data is provided for eighteen specimens – six series of three specimens each, produced with different angles of the steel bar axis to the vertical (build angle *b-a*) and different angles between torch axis and steel bar axis (nozzle angle *n-a*). Table 1 gives an overview of the specimen series, while the sketches in the diagrams from Fig. 1 illustrate the different angles.

**Table 1:** Overview of the specimen series.

Test series*	TS01	TS03	TS05	TS07	TS09	TS11
Nozzle angle (*n-a*)	0°	22.5°	45°	0°	22.5°	45°
Build angle (*b-a*)	0°	0°	0°	45°	45°	45°
Specimen names	TS01a TS01b TS01c	TS03a TS03b TS03c	TS05a TS05b TS05c	TS07a TS07b TS07c	TS09a TS09b TS09c	TS11a TS11b TS11c

⁎ The odd-numbered specimens mentioned in this table had as-printed irregular geometry. For these specimens, also geometrically and materially nonlinear analyses with imperfect geometry (GMNIAs) were conducted. The corresponding even-numbered specimens had milled surfaces (constant round cross-sections with regular surfaces). They were intentionally not included in the table and the data article, since no GMNIAs were conducted for them.

The odd-numbered specimens for which data is provided had as-printed irregular geometry. The corresponding even-numbered specimens had milled surfaces and were used in [1] for deriving an elastic-plastic material model for the wire arc additively manufactured carbon steel.

The dataset [2] described in this article consists of six files.

The ZIP-file *“01_scanned-geometry.zip”* contains 36 files – one STL-file and one CSV-file for each specimen. The files are named with the specimen name (e.g., *“TS01a.stl”* and *“TS01a.csv”* for the specimen TS01a). The STL-files contain the raw irregular triangular meshes obtained through 3D scanning. The CSV-files contain three columns with the coordinates in millimetres of the points with which the triangular meshes are built.

The CSV-file *“02_force-vs-displacement_experiments.csv”* contains the measured forces and displacements from the uniaxial tensile tests for all eighteen specimens. The forces are given in [kN] and the displacements in [mm]. The displacements are provided as elongations of a 38 mm long part of the wire arc additively manufactured steel bars. The file consists of three header rows specifying the specimen name, the type of data (displacement or force) and the units, respectively, followed by the rows with the data. The file consists of 36 columns, for each of the 18 specimens one with displacement values and one with force values.

The GH-file *“03_GH-script_Scan-mesh_to_FE-mesh.gh”* contains a Grasshopper script that was used to cut a 38 mm long part from the 3D scanned wire arc additively manufactured steel bars, to transform the irregular triangular meshes to more regular quad meshes, and to generate from these meshes closed surfaces that can be used for the finite element simulations. The script requires as input one of the STL-files from the ZIP-file *“01_scanned-geometry.zip”* and offers as output a closed surface that can then be exported for example from Rhino 3D as STP-file (see ZIP-file *“04_processed-geometry.zip”*).

The ZIP-file *“04_processed-geometry.zip”* contains 54 files – one STL-file, one CSV-file and one STP-file for each specimen. The files are named with the specimen name followed by an underscore and the letters “FE” (e.g., *“TS01a_FE.stl”, “TS01a_FE.csv”* and *“TS01a_FE.stp”* for the specimen TS01a). The STL-files contain the processed more regular quad meshes of the 38 mm long steel bar parts obtained from the irregular triangular 3D scanned meshes with the Grasshopper script *“03_GH-script_Scan-mesh_to_FE-mesh.gh”*. The CSV-files contain three columns with the coordinates in millimetres of the points with which the more regular quad meshes are built. The STP-files contain the closed surfaces that envelope the 38 mm long parts of the wire arc additively manufactured bars and which were imported to the computer aided engineering software Abaqus for performing the finite element simulations of the uniaxial tensile tests.

The ZIP-file *“05_input-files_simulations.zip”* contains 18 files – one INP-file for each specimen. The files are named with the specimen name (e.g., *“TS01a.inp”* for the specimen TS01a). The finite element simulations of the uniaxial tensile tests can be started from these input files. They include all the necessary information as geometry of the specimens, finite element mesh, boundary conditions, loading, requested data output intervals.

The CSV-file *“06_force-vs-displacement_simulations.csv”* contains the forces and displacements from the simulations of the uniaxial tensile tests for all eighteen specimens. The forces are given in [kN] and the displacements in [mm]. The displacements are provided as elongations of the 38 mm long parts of the wire arc additively manufactured steel bars used in the simulations. The file consists of three header rows specifying the specimen name, the type of data (displacement or force) and the units, respectively, followed by the rows with the data. The file consists of 36 columns, for each of the 18 specimens one with displacement values and one with force values.

For better understanding the provided data, Fig. 1 illustrates the data in CSV-file *“02_force-vs-displacement_experiments.csv”* and CSV-file *“06_force-vs-displacement_simulations.csv”* as curves for the different test specimens listed in Table 1.

## 4 Experimental Design, Materials and Methods

The data described in this article includes geometry information for wire arc additively manufactured (WAAM) steel bars and force-displacement data pairs obtained for such bars under tensile loading from experiments and finite element simulations. The production of the steel bars involved utilizing a configuration that included an ABB IRB 4600/40 robot, a Fronius TPS 500i Pulse power source, and a Fronius 60i Robacta Drive Cold Metal Transfer (CMT) torch featuring a 22° neck. A detailed description of the manufacturing process along with the used WAAM process parameters can be found in [1]. Steel bars with a target diameter of 8 mm and a length of 160 mm were printed. Different angles of the steel bar axis to the vertical (build angle *b-a*) and different angles between torch axis and steel bar axis (nozzle angle *n-a*) were used as illustrated by the sketches in the diagrams from Fig. 1. The bars were printed with the material Union SG 2-H [3]. This is a solid wire designed for the gas metal arc welding (GMAW) of unalloyed and low-alloy steels.

**Fig. 2:**
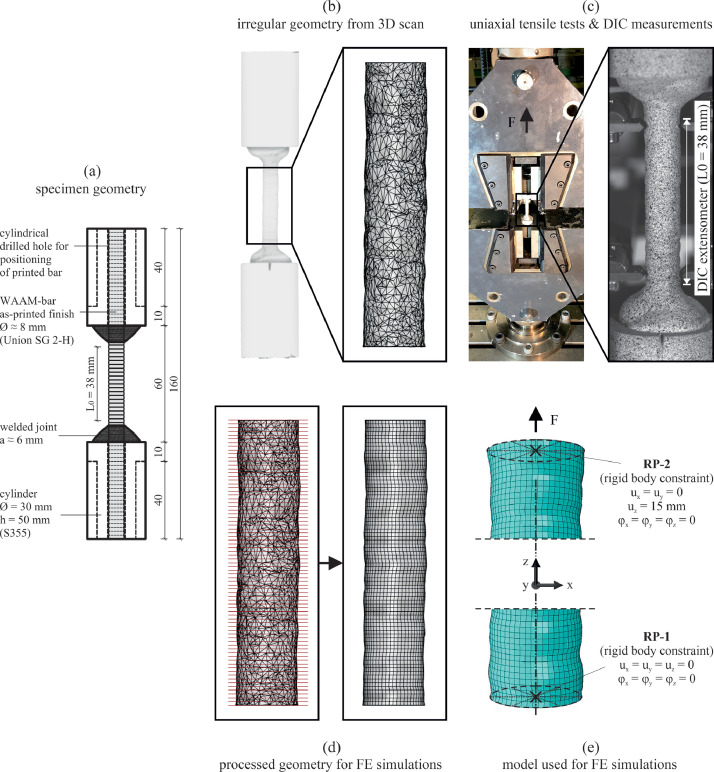
Methods applied for collecting the data described in the article: (a) uniaxial tensile test specimen geometry, (b) geometry obtained from 3D scanning, (c) setup and method for obtaining force-displacement data from uniaxial tensile tests, (d) geometry used for the finite element simulations, and (e) model for obtaining force-displacement data from finite element simulations.

The uniaxial tensile test specimens were designed based on specifications from EN ISO 6892-1 [4] and DIN 50125 [5], under consideration of particularities related to the production process by wire arc additive manufacturing. The additively manufactured steel bars were welded by robot into cylinders made of structural steel with a length of 500 mm, an outer diameter of 30 mm and a centrally drilled hole with a diameter of 10 mm. The geometry of the specimens is illustrated in Fig. 2a. The added structural steel cylinders *(i)* facilitated the 3D scanning process for obtaining the specimen geometry and *(ii)* allowed to minimize the risk for failure outside the desired measurement length for elongations.

The geometry of the wire arc additively manufactured steel bars exhibited strongly irregular surfaces due to the production process of welding droplet by droplet. The 3D scanner ATOS Core from GOM and the corresponding scan software, which operate based on principles of photogrammetry, were used for obtaining a 3D point cloud and a 3D triangular mesh of each specimen (see Fig. 2b). To conduct the measurements, a nearly imperceptible coating of matt white paint was applied to the WAAM steel bars to prevent reflections. Additionally, markers were affixed to the cylinders to facilitate image correlation among the roughly 30 pictures taken for each specimen from various perspectives. For the geometry data in the file *“01_scanned-geometry.zip”* from the shared dataset [2], the 3D scanned irregular triangular meshes were aligned in space with the software GOM Inspect [6]. The z-axis corresponded to the average of the axes of the two steel cylinders. The xy-plane was given by the top surface of the bottom cylinder on which a notch was cut with a milling tool. The positive x-axis was defined in the direction of this notch.

For the uniaxial tensile tests, a Zwick universal testing machine for loads up to 200 kN was used. The tests were conducted under displacement control with a displacement rate of 0.01 mm/s, which corresponds to a strain rate of 0.00025 s^−1^ ± 20% according to [4]. The test specimens were clamped in the machine on both sides over a length of 40 mm of the steel cylinders. The force was measured with a load cell, while for the displacement a digital image correlation (DIC) system from Correlated Solutions, Inc. with two cameras with a resolution of 12 MP was used. For the DIC measurements, an irregular speckle pattern of black ink dots on a matt white paint was applied on the specimens. The images were captured and evaluated with the VIC-3D 8 system from Correlated Solutions, Inc. For the displacements provided in the file *“02_force-vs-displacement_experiments.csv”* from the shared dataset [2], a virtual extensometer was applied on the steel bars within the VIC-3D 8 system over a length of 38 mm (see Fig. 2c) with which the elongation of this specimen part was calculated.

Geometrically and materially nonlinear analyses with imperfect geometry (GMNIA) were performed for the eighteen WAAM steel bars previously tested experimentally. For the simulations, only the 38 mm long part of the specimens was used, within which the failure occurred in the experiments and for which the elongation was calculated based on the DIC measurements. Since the very detailed irregular triangular mesh obtained by 3D scanning the specimens was not suitable for generating a finite element mesh, a Grasshopper script was used within the Rhinoceros 3D [7] environment to approximate it with a more regular quad mesh (see Fig. 2d). The Grasshopper script is provided in the file *“03_GH-script_Scan-mesh_to_FE-mesh.gh”*. It first cuts out the middle 38 mm long part of the 3D scanned specimen geometry and then regenerates the irregular surface with a quad mesh defined by section curves and a refinement factor of 0.5 mm. This refinement factor controls the distance between the section curves as well as the interval for segmentation of these section curves. Finally, the script transforms the quad mesh into a closed surface which can be baked from Grasshopper into Rhinoceros 3D and from there exported as STP-file.

The GMNIA simulations were performed with the computer aided engineering software Abaqus [8]. The geometry of the specimens was imported from the STP-files previously generated with the Grasshopper script in the Rhinoceros 3D environment. An overview of the model showing the finite element mesh density, the boundary conditions and the loading is shown in Fig. 2e. A finite element mesh size of 0.5 mm was chosen based on a convergence study. The study aimed at using an as coarse as possible mesh size to allow a shorter computation time, but at the same time an as fine enough mesh as necessary for reproducing the irregular surfaces accurately enough in order to correctly predict the failure points along the bar length. Mesh size values that are a divisor of the considered bar length (38 mm), print layer height (1 mm) and section-curves refinement factor for generating the geometry (0.5 mm) were considered. Twenty-node quadratic brick elements with reduced integration (C3D20R) were used. These showed a slightly better suitability, especially for reproducing the behaviour of the test specimens in the necking part after reaching the maximum force, compared to the 8-node linear brick elements with reduced integration (C3D8R), which were used for the simulations in [1]. Boundary conditions and loading were imposed on two reference points linked to the terminal surfaces of the 38 mm long WAAM bar components, employing rigid body constraints. For the bottom reference point (RP-1), all translational and rotational degrees of freedom were restrained, while for the top one (RP-2) all rotational degrees of freedom and the two translational degrees of freedom perpendicular to the axis of the bars were fixed. The loading was applied as a displacement of 15 mm on the top reference point (RP-2) in positive direction of the bar axis (z-direction). Two loading steps were defined, one until 2.5 mm and the other one until 15 mm displacement. For each of these steps 125 data pairs of force and displacement were requested as output. The material model used in the simulations was an elastic-plastic one, derived from uniaxial tests on milled WAAM steel bars, as described in [1]. For the elastic properties, a Young's modulus of 195,000 MPa and a Poisson's ratio of 0.3 were defined. Regarding the plastic properties, ten sets of data, consisting of true yield stress and true plastic strain pairs, were specified, as outlined in Table 2. The model definitions described here can be found for each of the eighteen simulated specimens in the Abaqus input files provided in the file *“05_input-files_simulations.zip”*.

**Table 2:** Plastic material properties for the WAAM steel as true yield stress values at true plastic strains.

True yield stress σ_y,true_ [MPa]	360.0	374.1	442.2	520.6	569.2	606.4	706.3	772.2	864.0	944.6
True plastic strain ε_pl,true_ [-]	0.000	0.028	0.050	0.100	0.150	0.200	0.400	0.600	1.000	1.500

**Fig. 3:**
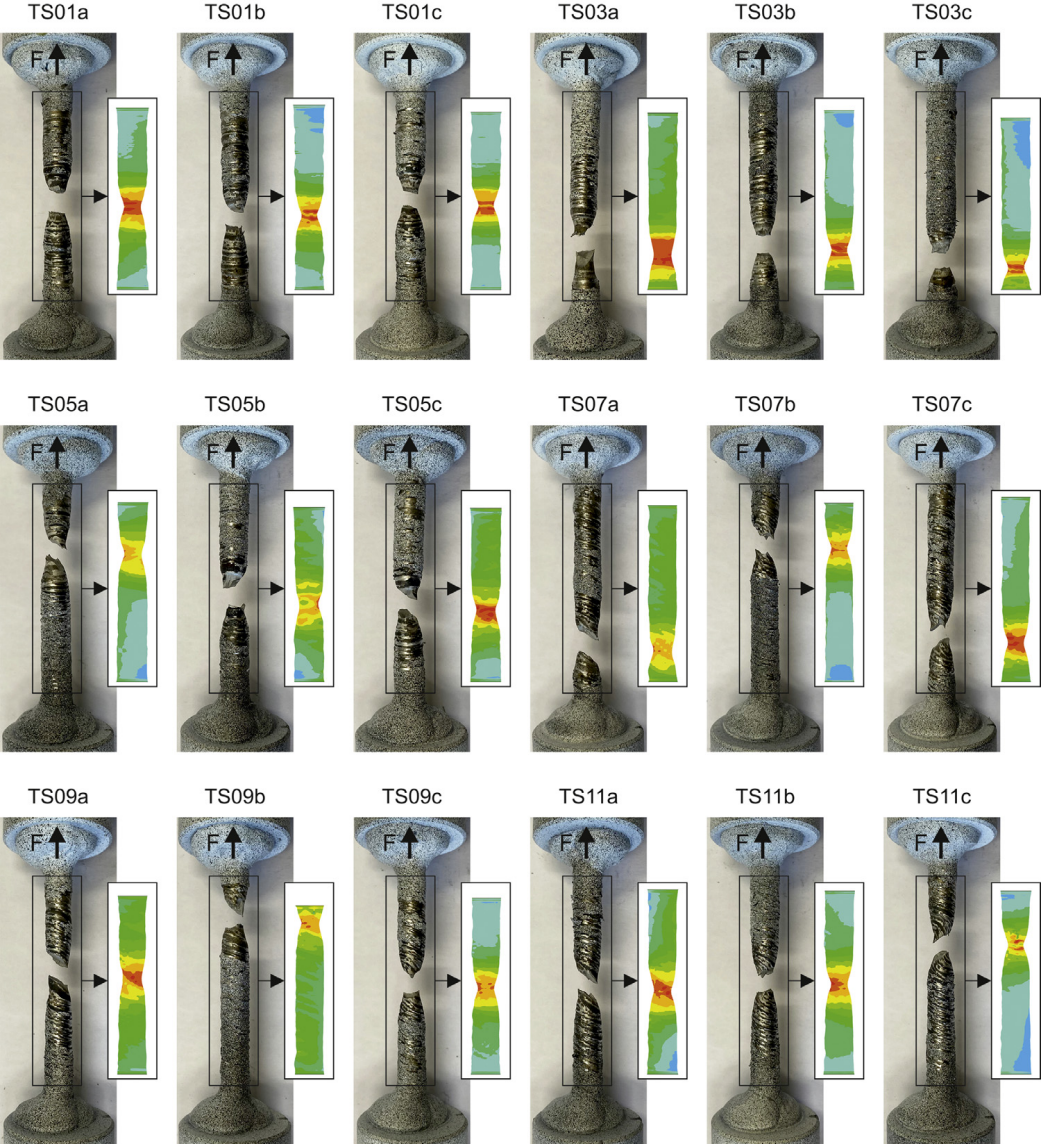
Uniaxial tensile test specimens after failure and corresponding qualitative stress contour plots from the GMNIA simulations.

For better understanding the performed experimental tests and finite element simulations as well as the provided data, Fig. 3 illustrates pictures of the 18 specimens after failure in the uniaxial tensile tests and corresponding qualitative stress contour plots from the performed geometrically and materially non-linear analyses with imperfect geometry (GMNIA). The stress contour plots from the simulations were printed for the same force at which the failure occurred in the uniaxial tensile experiments after necking, not for the same displacement (see force versus displacement curves in Fig. 1; the shown contour plots are for the force values at which the test curves end or show an almost vertical drop).

## Limitations

There are no significant limitations for the data described in this article regarding data collection and curation. The only aspect worth mentioning is that the orientation of the wire arc additively manufactured steel bars was only tracked starting with the 3D scanning of the geometry. This means that the steel bars were oriented in the same direction for all the methods applied to collect the data described in the article (3D scanning of imperfect geometry, uniaxial tensile tests, and finite element simulations). However, a correlation of the bottom and top ends of the steel bars during manufacturing and their orientation during the subsequent steps was not carried out. However, this has no relevance for the data described in the current article, only eventually for the geometry data evaluated in [1].

## Ethics Statement

The authors have read and follow the ethical requirements for publication in Data in Brief and confirm that the current work does not involve human subjects, animal experiments, or any data collected from social media platforms.

## CRediT authorship contribution statement

**Vlad-Alexandru Silvestru:** Conceptualization, Methodology, Software, Validation, Formal analysis, Investigation, Data curation, Writing – original draft, Writing – review & editing, Project administration.

## Data Availability

Dataset for point-by-point wire arc additively manufactured carbon steel bars loaded in uniaxial tension - experiments and simulations (Original data) (ETH Zurich Research Collection)
